# Directionality of Interpersonal Neural Influence in Functional Near‐Infrared Spectroscopy Hyperscanning: Feasibility of Information–Theoretic Causality Analysis in Motor Tasks

**DOI:** 10.1111/ejn.70252

**Published:** 2025-09-24

**Authors:** Maha Shadaydeh, Vanessa Noering, Marcel Franz, Tara Chand, Ilona Croy, Joachim Denzler

**Affiliations:** ^1^ Department of Mathematics and Computer Science Friedrich Schiller University Jena Jena Germany; ^2^ Department of Clinical Psychology, Institute of Psychology Friedrich Schiller University Jena Jena Germany; ^3^ German Center for Mental Health Halle‐Jena Magdeburg Germany; ^4^ Department of Humanistic Studies Indian Institute of Technology Varanasi India

**Keywords:** fNIRS, hyperscanning, imaging, interpersonal synchronization, mutual information decomposition, social interaction, spectral causality

## Abstract

Hyperscanning approaches mark a shift from single‐ to two‐person neuroscience, enabling a more profound understanding of the neural mechanisms underlying interpersonal synchronization. In this context, functional near‐infrared spectroscopy (fNIRS) has emerged as a valuable tool for measuring brain activity in a natural, unconstrained environment. While interpersonal synchrony using fNIRS hyperscanning has been well‐studied using statistical association analysis, establishing causal relationships that elucidate the direction of influence remains challenging. This study aimed to investigate the feasibility of determining the direction of influence in dyadic interactions. Since the ground truth of such direction is not available in a natural setting, we validated our approach in an experimental setup in which we controlled the direction of influence between two subjects by assigning them the roles of “Model” and “Imitator” of specified motor tasks. A total of 22 participants, hence 11 dyads, completed the task in a within‐subject design. We adapted concepts from spectral causal effect decomposition theories to formulate a new measure of the direction and intensity of influence. The results demonstrate that the direction of influence in fNIRS data can be detected with an accuracy in the range of 62%–71%. Furthermore, the proposed spectral causality measure was shown to significantly reduce spurious causal relationships due to the confounding effects of physiological processes and measurement artifacts compared to time domain causal analysis.

## Graphical abstract

This study aimed to investigate the feasibility of testing the direction of influence in functional near‐infrared spectroscopy (fNIRS) hyperscanning. Validation is performed on an experimental setup with a controlled direction of influence in motor tasks. The proposed spectral causality measure significantly reduced the confounding effects of physiological and measurement noise, reaching 62%–71% accuracy.

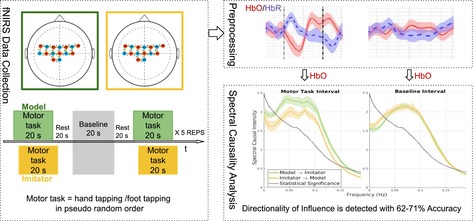

## 1 Introduction

Hyperscanning involves the simultaneous recording of brain activity from two or more individuals to determine the temporal relation between both brains (synchronization). Hyperscanning approaches thereby mark a shift from single‐ to two‐person neuroscience, allowing a much deeper understanding of the neural mechanisms of interpersonal social interactions (Czeszumski et al. 2020). Such research revealed synchronized patterns of brain signals in interacting minds, especially in brain regions involved in social cognition, emotion, and motor control (for an overview, see (De Felice et al. 2025)).

Different hyperscanning measurements such as electroencephalograph (EEG), functional magnetic resonance imaging (fMRI), and functional near‐infrared spectroscopy (fNIRS) have been used to investigate interpersonal synchronization during verbal, semiverbal, and nonverbal interactions (Hakim et al. 2023). Compared to fMRI and EEG, fNIRS offers significant advantages for monitoring neural activity during natural, unconstrained, and real‐life interactions. Its high temporal resolution of oxygenation change and motion tolerance makes it particularly valuable for capturing dynamic neural activity in naturalistic settings (Pinti et al. 2020).

While interpersonal synchrony using fNIRS hyperscanning has been well studied using statistical association analysis, for example, temporal correlation (Han et al. 2022) or wavelet coherence (Nguyen et al. 2021), establishing causal relationships that elucidate the direction of influence in hyperscanning remains challenging (for an overview, see (Hakim et al. 2023)). Therefore, this study aimed to advance beyond direction‐blind statistical association to investigate the feasibility of testing the direction of influence in dyadic interactions using causal analysis methods.

A central and ongoing debate in the hyperscanning literature concerns the causal role of interbrain synchrony (IBS): Do brains that synchronize during interaction merely reflect shared sensory input and task structure, or does IBS itself facilitate social interaction? While early studies focused on demonstrating the presence of IBS during joint tasks (Liu et al. 2016; Nguyen et al. 2020), a recent debate in the field revolves around understanding the source and functional significance of IBS (Holroyd 2022); in this sense, some authors (Novembre and Iannetti 2021) have questioned whether this synchrony is epiphenomenal or mechanistically relevant for social behavior. Novembre and Iannetti (Novembre and Iannetti 2021), for instance, argue that hyperscanning, due to its correlational nature, cannot resolve this question. They propose that only interventional approaches, such as dual‐brain stimulation (DBS), can provide direct causal evidence by actively manipulating IBS and observing its effects on social behavior. This study aims to contribute to this debate by exploring whether causal discovery methods applied to fNIRS hyperscanning data can reveal directional dependencies between interacting brains. Our work thus complements the broader effort to move from correlation to causation in social neuroscience by providing a data‐driven framework for inferring directional interbrain effects in naturalistic settings.

Causal discovery in multivariate time series aims to elucidate the cause‐and‐effect relationships between variables that evolve over time. The most well‐known classical method is the Granger causality (GC) (Granger 1969). GC is based on the idea that causes precede and help predict their effects. Recent research has increasingly focused on understanding the synergistic effects of groups of variables acting as a collective subsystem on other groups. This focus is particularly critical in complex systems characterized by intricate interdependencies, such as climate–ecosystem interactions and neural activity across distinct brain regions of the same subject (Faes et al. 2022). Notable group causality methods are the Trace method (Zscheischler et al. 2011), the 2GVecCI (Wahl et al. 2023), Vanilla‐PC (Janzing et al. 2009), and MC‐VAR (Ashrafulla et al. 2013). While these methods operate in the time domain, Faes et al. (Faes et al. 2022) built on the spectral causality approach of Geweke (Geweke 1982) and proposed an information–theoretic framework based on mutual information rate (MIR) decomposition to assess the interactions among groups of processes, both within specific frequency bands of interest and in the time domain.

fNIRS data are often influenced by various sources of noise stemming from measurements and physiological processes, for example, breathing, heart rate, and Mayer waves, (Pinti et al. 2019). In hyperscanning, these processes typically occur at similar frequency ranges in both participants, thus can confound the results, leading to spurious associations between participants when using time domain statistical or causal analysis. Furthermore, the strength of coupling may vary across different frequency bands. To address these challenges, we adapted the framework of Faes et al. (Faes et al. 2022), to our specific research question, subsequently proposing a new measure for quantifying the direction and intensity of causal effect relationships in fNIRS data. Since the ground truth of the direction of interpersonal influence is not available in a natural setting, we validated our approach in an experimental setup where we controlled the direction of influence between two subjects. We compared the results of different state‐of‐the‐art group causality methods to the proposed spectral domain causal effect measure. We showed the feasibility of detecting the correct cause–effect direction in fNIRS time series data. To our knowledge, this paper is the first to provide a comprehensive analysis pipeline for identifying the direction of influence in fNIRS data.

## 2 Materials and Methods

### 2.1 Participants

A total of 11 dyads, 22 participants, were recruited from the student population, with a mean age of 23.15 and a standard deviation of 2.58. The sample was 21 females and one male. Inclusion criteria required participants to be at least 18 years old and report to be neurologically healthy. Participants received research participation credits as compensation. The study was conducted following the Declaration of Helsinki and approved by the ethics review board of the Faculty of Social and Behavioral Sciences of the University of Jena (FSV 22/063).

### 2.2 Experimental Design

Participants were invited in dyads to perform a dyadic movement imitation task. Initially, each person was assigned to either the role of Model (M) or Imitator (I). Both participants were seated opposite each other, so the Model faced a screen behind the Imitator, which was invisible to the Imitator. We presented two 20‐s videos on the screen, one showing hand‐tapping and the other foot‐tapping. For hand‐tapping, the video showed a person's hand with each finger (excluding the thumb) sequentially tapping on a surface at a rate of approximately 1.5 Hz. For the foot‐tapping task, the video showed a barefoot‐tapping on the floor at the same rate. The Model's task was to watch the screen and copy the movement with their right hand or foot. The Imitator's task was to imitate the movement of the Model.

A fixation cross was displayed for 60 s before each video, serving as a rest and baseline during which participants were asked not to move. Videos were presented in a pseudo‐randomized order five times each, resulting in ten trials per Model–Imitator constellation. After a short break, the Model and Imitator switched roles and repeated the experiment with a different stimulus order. The experiment and the video presentation were programmed and controlled using the Presentation software (Version 23.0, Neurobehavioral Systems, Inc., Berkley, CA).

### 2.3 Data Acquisition

**FIGURE 1:**
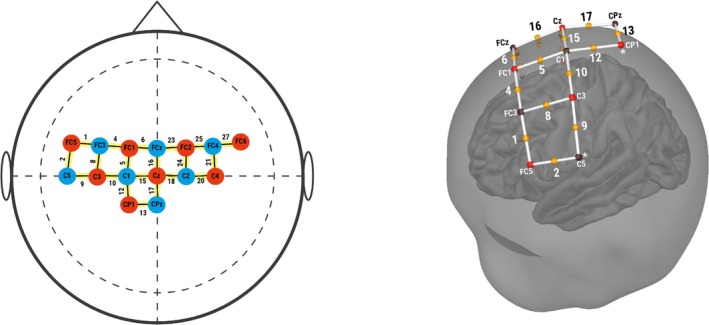
The optodes layout used for fNIRS measurements (2D and 3D views). The brain motor regions are M1 (Channels 12, 13, and 17), PMC (Channels 4, 5, 6, 8, and 16), and PMC/M1 (Channels 10 and 15).

Each participant's cortical hemodynamic activity was recorded using a continuous wave fNIRS system (NIRSport2, NIRx, Germany) with a sampling frequency $f_{s}=10.17$ Hz and 15 optodes per participant (eight emitters × 7 detectors). Based on a finger‐ and foot‐tapping study by Cockx et al. (Cockx et al. 2023), the optodes were placed to cover the left and right primary motor cortex (M1) and premotor cortex (PMC) (Figure 1) with a distance of 3 cm to allow measurement of cerebral blood oxygenation at 2‐ to 3‐cm depth. Additionally, eight short‐distance channels (SDCs) were placed at each emitter position for later offline short‐channel correction of nonneuronal signals from long‐channel data.

### 2.4 Data Preprocessing

**FIGURE A1:**
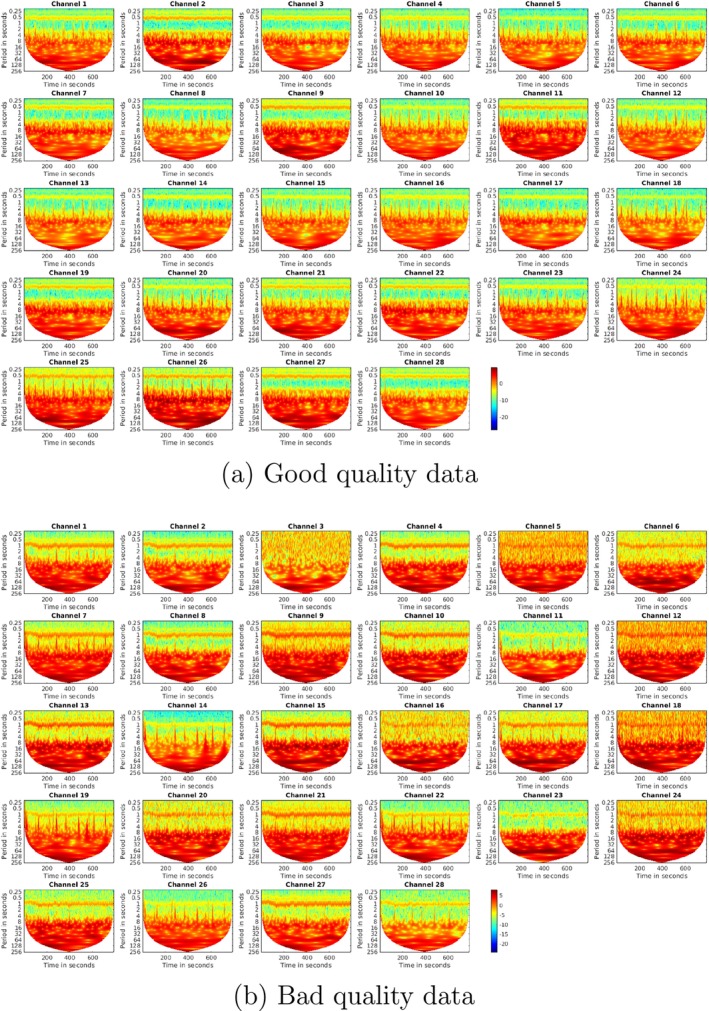
fNIRS data visual quality check using Wavelet transform plots before any filtering: (a) a good quality data with a clear separation between the heart rate at the period of ≈1sec and the lower frequencies (higher periods) and (b) a bad quality data where high wavelet values occur at all frequencies and all time samples.

The preprocessing of the fNIRS time series was performed using the Homer3 MATLAB toolbox (Huppert et al. 2009). The raw optical intensity time series for every subject were converted into changes in optical density (OD) using the hmrR_Intensity2OD function. To detect motion artifacts, the hmrMotionArtifactByChannel was applied to the OD time series with the parameters: AMPthresh=1, SDThresh=10, tMotion = 1, and tMask = 1. We used the Spline correction function hmrR_MotionCorrectSpline with p=0.99 (Scholkmann et al. 2010) for motion artifact correction. Hemoglobin concentration changes (ΔHb) were estimated using the modified Beer–Lambert law with the hmrR_OD2Conc function. A supplementary wavelet‐based visual quality control procedure (Nguyen et al. 2021) was implemented before the filtering process. Figure A1 shows examples of good and bad quality HbO signals. Ten of the 11 dyads exhibited good data quality and were included in the subsequent analysis. Finally, using the hmrR_BandpassFilt function, a fifth‐order Butterworth bandpass filter was applied to ΔHb with a low cutoff frequency of 0.008 Hz and a high cutoff frequency of 0.2 Hz. The phase of the used filter is almost linear in the passband; that is, all signal components undergo a similar delay, and thus, no influence on causal analysis is expected due to this filtering process. This filtering step removes physiological noise, such as respiratory fluctuations (≈0.25 Hz), cardiac oscillations (≈1 Hz) (Hakim et al. 2023), and slow drifts in the baseline signal, while preserving neural activity in the typical frequency range of interest (≈0.029 Hz, depending on the stimulus presentation rate (Hakim et al. 2023)).

**FIGURE 2:**
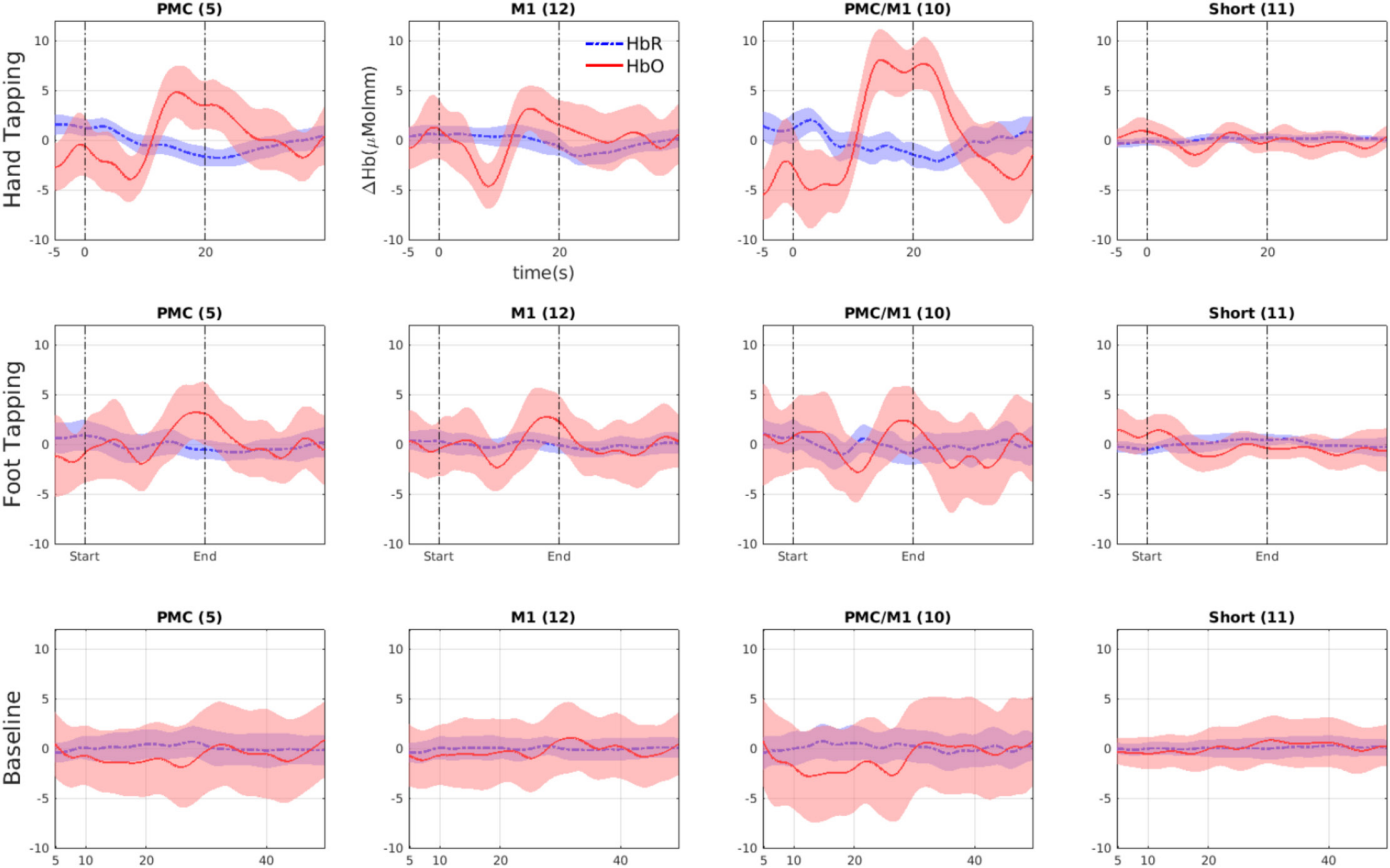
The average and 50*%* confidence interval of the oxy‐/de‐oxygenated hemoglobin time series (HbO/HbR) in red/blue color. The time series are averaged for each channel over all dyads and task repetitions for hand‐tapping, foot‐tapping, and baseline tasks. The start and end of the video on the Model's screen in the motor task intervals are marked in vertical lines; the task is performed for 20 seconds.

The averages and variances of the oxygenated hemoglobin (HbO) and deoxygenated hemoglobin (HbR) were modelled using the block averaging functions of Homer3. Figure 2 illustrates these signals for hand‐tapping, foot tapping, and baseline intervals in a sample channel in each brain motor region: M1, PMC, and PMC/M1. The start line is the start of the video on the screen visible to the Model only. We show the HbO average for the Imitators in the second five trials, where both participants became more familiar with the task. We can see an apparent increase in HbO signals during motor tasks compared to the baseline condition. Residual periodic fluctuations, likely attributed to Mayer waves, are observable at ≈0.1 Hz (≈ two waves in 20 s). We calculated the correlation between HbO and HbR to support our claim that the signal is related to neural activity. This negative correlation can also be seen in Figure 2 from the synchronization between the increase of HbO and the decrease of HbR in all channels.

The temporal profile of the hemodynamic response (HbO) is comparable in shape and peak location to the findings reported in (Cockx et al. 2023). However, the onset of activation in the present study appears delayed by approximately 2–3 s. This delay can be attributed to the definition of the time axis, where time zero corresponds to the start of the video displaying the target movement. Participants typically initiated their motor response 2–3 s after video onset, which plausibly explains the observed temporal shift. This delay of the peak is also likely influenced by our task duration being 20 s, compared to the 8 s duration in Cock et al. (Cockx et al. 2023).

Since our target is to detect the cause‐effect relationship, we avoided any nonlinear processing of the signals, such as GLM modeling or the use of SDCs to further clean data. Our primary goal was to preserve the temporal relationship between the Model and the Imitator since any nonlinear processing could alter this crucial element of our analysis. The methods we selected for filtering, as explained above, and motion correction were chosen such that they do not change this temporal relationship, which could otherwise compromise the causality results. Our main proposal and recommendation of this paper is to work in the frequency domain to remove any causality related to motions or physiological artifacts by defining causality at the frequency band of interest. Nevertheless, the SDCs were used for further motion compensation in terms of causal intensities, as will be explained in the following section.

**FIGURE 3:**
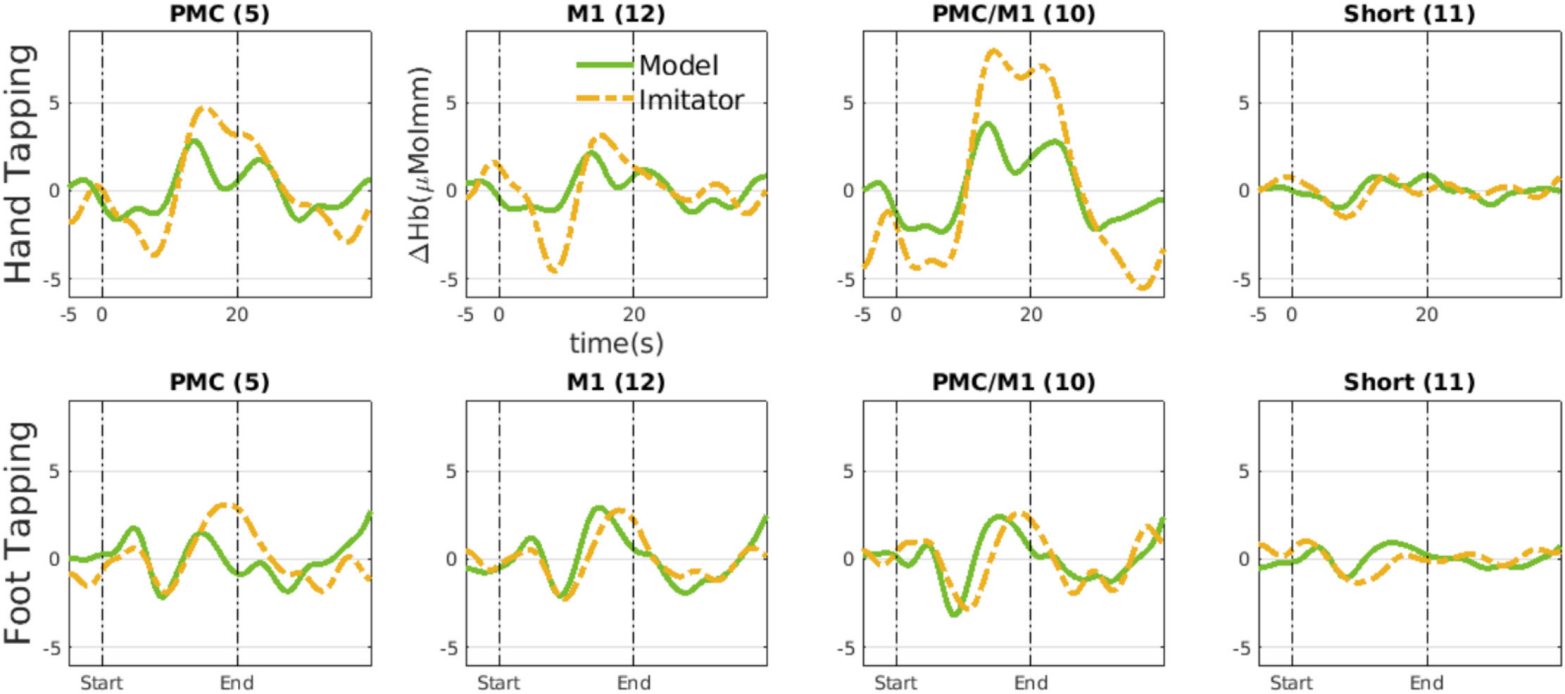
The average of the oxygenated hemoglobin time series (HbO) for the Model (in green) and Imitator (in yellow). The delay between Model and Imitator is larger for the foot tapping task than for the hand‐tapping task. Different regions exhibited different delays between the Model and the Imitator.

**FIGURE B1:**
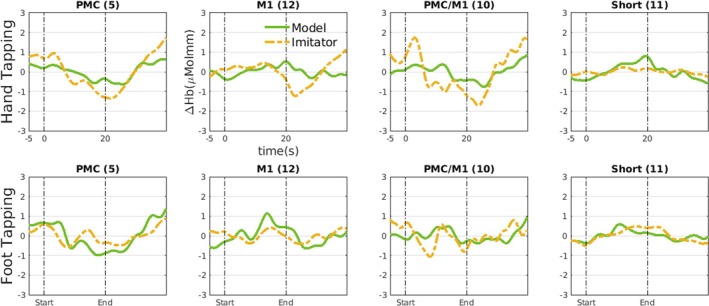
The average of the deoxygenated hemoglobin time series (HbR) for the Model (in green) and Imitator (in yellow). There is no consistent pattern for the delay between the Model and the Imitator.

Figure 3 shows the temporal relation between the Model and Imitator for the HbO signals. We can notice that the activation of the Imitator's HbO follows the HbO activation of the Model. This clear delay links to the concept of GC in the temporal sense that the cause precedes and helps predict the effect. Interestingly, we can also notice how this delay differs in different tasks and regions. The delay is larger in the foot tapping task than in the hand‐tapping task. In contrast, for the HbR signals, this temporal relation between the Model and Imitator is no longer consistent, as shown in Figure B1.

### 2.5 The Directionality of Neural Influence: A Spectral Causality Approach

To identify the direction of influence between the two participants (Model and Imitator) within each dyad, we adapted the Spectral Decomposition of MIR framework of (Geweke 1982; Faes et al. 2022), hereafter referred to as spectral MIR (Spectr‐MIR). In the following, we first provide a brief overview of the Spectr‐MIR method adapted to our problem. We subsequently propose our definition of the measure quantifying the intensity and direction of the causal effect between the Model and the Imitator, along with the statistical significance test used.

#### 2.5.1 Spectr‐MIR Method

Let $X(t_{n})\in {\mathbb{R}}^{L\times 2N}$ be L×2N matrix representing N time series of length L of a specific brain region for both the Model (M) and Imitator (I), respectively, where $t_{n}=n\Delta t$ is the time index in iteration n and $\Delta t=1/f_{s}$. The matrix $X(t_{n})$ can be represented as the concatenation of the HbO channels of the Model $X_{M}$ and Imitator $X_{I}$, that is, $X(t_{n})=[X_{M}(t_{n})\;X_{I}(t_{n})]$. The information shared by the two random processes $X_{M}(t_{n})$ and $X_{I}(t_{n})$ per unit of time is defined as the MIR as follows (Duncan 1970):

(1) $$\begin{array}{c} {\text{MIR}}_{X_{M};X_{I}}=\underset{k\to \infty }{\lim}\frac{1}{k}\text{MI}\left(X_{M}(t_{n-k:n-1});X_{I}(t_{n-k:n-1})\right), \end{array}$$

where $\text{MI}(X_{1},X_{2})$ denotes the mutual information (MI) shared by the two variables $X_{1}$ and $X_{2}$ which is defined as follows:

(2) $$\text{MI}(X_{1};X_{2})=\mathrm{34𝔼}\left[\log\frac{p(x_{1},x_{2})}{p(x_{2})p(x_{1})}\right].$$

Here, p(.,.) and p(.) denote joint and marginal probabilities, and 34𝔼 is the statistical expectation operator. Using the relation between transfer entropy and MI, it is possible to decompose the MIR into three components, that is (Faes et al. 2022; Duncan 1970),

(3) $${\text{MIR}}_{X_{M};X_{I}}=T_{X_{M}\to X_{I}}+T_{X_{I}\to X_{M}}+{\text{MIR}}_{X_{M}.X_{I}}.$$

${\text{MIR}}_{X_{M}.X_{I}}$ represents the instantaneous information shared between $X_{M}$ and $X_{I}$ and $T_{X_{1}\to X_{2}}$ is the entropy transfer from $X_{1}$ to $X_{2}$.

Following the methodology of (Duncan 1970; Faes et al. 2022), we utilize a state‐space modeling approach to compute all necessary MIR terms. Accordingly, we present the process $X(t_{n})$ as a state‐space model, that is,

(4) $$\begin{array}{cc} S(t_{n+1})\; & =AS(t_{n})+KW(t_{n}),\\ X(t_{n})\; & =CS(t_{n})+W(t_{n}). \end{array}$$

$S(t_{n})$ is the 2N×p state vector of the model, where p is the model order; A,C, and K are the state‐space model matrices, and $W(t_{n})$ is a white Gaussian innovation noise vector of zero mean and covariance matrix $\Sigma_{W}=\mathrm{34𝔼}[W_{n}W_{n}^{T}]$. Similar to $X(t_{n})$, $W(t_{n})$ also can be written as $W(t_{n})=[W_{M}(t_{n})\;W_{I}(t_{n})]$.

Taking the Fourier transform (FT) of the state Equation (4) yields

(5) $$S(\omega )=AS(\omega )e^{-j\omega }+KW(\omega )e^{-j\omega },$$

where S(ω) and W(ω) are, respectively, the FTs of $S(t_{n})$ and $W(t_{n})$ and ω is the normalized angular frequency. From Equation (5), we can derive the power spectral density (PSD) of $X(t_{n})$ as X(ω)=H(ω)W(ω), where

(6) $$H(\omega )=\left(I_{2N\times p}+C{\left[I_{2N\times p}-Ae^{-j\omega }\right]}^{-1}Ke^{-j\omega }\right),$$

with I being the identity matrix. H(ω) represents the transfer function relating the FT of the innovation process $W(t_{n})$ to the FT of the process $X(t_{n})$ and can be used together with the innovation covariance matrix to derive the PSD matrix of the process $X(t_{n})$ using spectral factorization.

(7) $$S_{X}(\omega )=H(\omega )\Sigma_{W}H^{*}(\omega ).$$

The matrix $S_{X}(\omega )$ can be then factorized to get the power spectral densities of $X_{M}$ and $X_{I}$, $S_{X_{M}}(\omega )$ and $S_{X_{I}}(\omega )$ and the cross‐spectral densities between $X_{M}$ and $X_{I}$ and $S_{X_{M}X_{I}}(\omega )$ and $S_{X_{I}X_{M}}(\omega )$. A logarithmic spectral measure of the interdependence between $X_{M}$ and $X_{I}$ is defined by (Geweke 1982).

(8) $$f_{X_{I};X_{M}}(\omega )=\log\frac{\left|S_{X_{I}}(\omega )||S_{X_{M}}(\omega )\right|}{\left|S_{X}(\omega )\right|},$$

where $f_{X_{I};X_{M}}(\omega )$ is a measure of the total spectral coupling between $X_{I}$ and $X_{M}$, which, in analogy to the time domain decomposition, can be factorized into three components.

(9) $$f_{X_{I};X_{M}}(\omega )=f_{X_{I}\to X_{M}}(\omega )+f_{X_{M}\to X_{I}}(\omega )+f_{X_{I}.X_{M}}(\omega ),$$

where $f_{X_{1}\to X_{2}}(\omega )$ is a measure of the density of information transferred from process $X_{1}$ to process $X_{2}$ and $f_{X_{I}.X_{M}}(\omega )$ is the information shared between the two processes at angular frequency ω. These measures are defined as follows:

(10) $$f_{X_{I}.X_{M}}(\omega )=\log\frac{\left|H_{M}(\omega )\Sigma_{W_{M}}H_{M}^{*}(\omega )||H_{I}(\omega )\Sigma_{W_{I}}H_{I}^{*}(\omega )\right|}{\left|S_{X}(\omega )\right|},$$

(11) $$f_{X_{M}\to X_{I}}(\omega )=\log\frac{\left|S_{X_{I}}(\omega )\right|}{\left|H_{M}(\omega )\Sigma_{W_{M}}H_{M}^{*}(\omega )\right|},$$

(12) $$f_{X_{I}\to X_{M}}(\omega )=\log\frac{\left|S_{X_{M}}(\omega )\right|}{\left|H_{I}(\omega )\Sigma_{W_{I}}H_{I}^{*}(\omega )\right|}.$$

Here, $H_{\left(\cdot \right)}(\omega )$ describes the transfer from $W_{\left(\cdot \right)}$ to $X_{\left(\cdot \right)}$ in the frequency domain and $\Sigma_{W_{\left(\cdot \right)}}=\mathrm{34𝔼}[W_{(\cdot ),n}W_{(\cdot ),n}^{T}]$.

In our study, the state‐space model, as defined in Equation (4), represents only the channels of the two regions of interest in the Model and Imitator and not the channels of all regions in both participants. We justify our choice by arguing that we are only interested in the interdependencies of a specific brain region in both Model and Imitator, regardless of the intradependency of other regions in the same person's brain. Moreover, focusing on a specific brain region at a time can benefit from better model fitting due to lower dimensionality since the intervals of the motor task are only of size 200 samples, which is insufficient to accurately fit a higher dimensionality model.

#### 2.5.2 Spectral Causal Intensity Measure

Our objective was to measure the intensity and direction of the causal effect relationship between a specific region of interest (ROI) in the brain of the Model and the same ROI of the Imitator. Model and Imitator are, in principle, two independent entities. In our settings, any bidirectional causality and/or detected cause–effect during baseline intervals presumably results from some unobserved factor influencing both participants, such as a physiological process occurring at the same frequency range, task repetition frequency, or common noise occurring during signal measurement and acting as a confounder. To eliminate, as much as possible, any causality due to confounders, we propose to measure the causal effect of the Model on the Imitator in the frequency domain at frequency ω as follows:

(13) $$C_{X_{M},X_{I}}(\omega )=f_{X_{M}\to X_{I}}(\omega )-f_{X_{I}\to X_{M}}(\omega ).$$

#### 2.5.3 Statistical Significance of Spectr‐MIR

To assess the statistical significance of the causal relationships identified, we use a frequency domain surrogate data method (Theiler et al. 1992). This approach preserves the amplitude spectrum of the original HbO time series while randomizing the phase information, effectively breaking the temporal dependencies within the data. The following steps are applied to the HbO time series: 1. Compute the FT of the original HbO time series. 2. Replace the original phase of each Fourier coefficient with a random phase drawn from a uniform distribution between 0 and 2π. 3. Perform the inverse FT to produce a surrogate HbO time series. 4. Apply the same causal inference method described above to the generated surrogate HbO time series. This procedure is repeated several times to produce an ensemble of surrogate time series. The spectral causality value of the HbO time series data is considered significant at a specific frequency only if it exceeds the spectral causality of the surrogate data at this frequency.

### 2.6 The Directionality of Neural Influence: Time Domain Causal Analysis

#### 2.6.1 MIR Method

The causal intensity and direction of the time domain Spectr‐MIR method, hereafter referred to as MIR, can be obtained for a specific ROI by the integration of $C_{X_{M},X_{I}}(\omega )$ over a specific band of frequencies from $\omega_{1}$ to $\omega_{2}$

(14) $$C_{X_{M}\to X_{I}}=\frac{1}{4\pi }\int_{\;\omega_{1}}^{\omega_{2}}C_{X_{M},X_{I}}(\omega )d\omega .$$

In our experiments, to reduce possible spurious causality due to motion as well as the superficial scalp activities captured by the SDC, we calculated the integral of Equation (14) in the range of 0.008–0.08Hz instead of the whole range, as motion‐related frequencies are more likely be in the higher part of the 0.008‐ to 0.2‐Hz range. We define, for each dyad and each of the ten trials of each task, the intensity of the causal effect as the absolute value of $C_{X_{M}\to X_{I}}$. The direction of the causal effect is from the Model $X_{M}$ to Imitator $X_{I}$ if $C_{X_{M},X_{I}}>C^{short}+\epsilon$, where ϵ is a very small positive value that defines the range of no causality and is set to 0.0001 in this study. Here, $C^{short}=mean(C_{Xs_{M}\to Xs_{I}})$, where $C_{Xs_{M}\to Xs_{I}}$ is the causal intensity from Model to Imitator calculated using the same Spectr‐MIR method on the SDCs of the left side of the brain, namely, channels (7, 11, and 14). If $C_{X_{M},X_{I}}<C^{short}-\epsilon$, the direction of influence is from Imitator $X_{I}$ to Model $X_{M}$. In simple terms, we assume that there is causality from Model to Imitator if the causal intensity from Model to Imitator of a specific ROI exceeds the average value of causality calculated from the SDC for each dyad over the 10 trials of this dyad. This definition supports the elimination of spurious causal influence between the Model and the Imitator due to motion and physiological processes that the filtering step of preprocessing could not eliminate.

#### 2.6.2 Time Domain Group Causality Methods

To assess the performance of the time domain causal direction estimation using the MIR method, we compare it with the following four state‐of‐the‐art time domain group causality methods.

‐ Vanilla‐PC (Janzing et al. 2009): A framework for inferring causal directions between groups of variables by applying a series of conditional independence tests.
‐ Trace (Zscheischler et al. 2011): This method infers whether linear relations between two high‐dimensional variables X and Y are due to a causal influence from X to Y or from Y to X.
‐ 2GVecCI (Wahl et al. 2023): A nonparametric approach for inferring the causal relationship between two vector‐valued random variables from observational data based on a series of conditional independence tests.
‐ Canonical GC (MC‐VAR) (Ashrafulla et al. 2013): This method combines ideas from canonical correlation and GC analysis to yield a measure that reflects directed causality between two regions of interest using optimized linear combinations of signals from each ROI to enable accurate causality measurements.

## 3 Results

### 3.1 Spectral Causal Analysis Results

To evaluate the performance of the Spectr‐MIR method in the frequency domain, we applied the method for each motor task and for each of the brain motor regions of interest, namely, M1 (Channels 12, 13, and 17), PMC (Channels 4, 5, 6, 8, and 16), and PMC/M1 (Channels 10 and 15) separately. We chose to work with an interval length of 204 samples, equal to the samples of the task interval (20s×10.17Hz≈204 samples), starting 1 s (10 samples) before the start of the video of the targeted movement. The state‐space model of Equation (4) is used to model the HbO time series in the brain ROI of both Model and Imitator for the 20‐s intervals of each task, each trial, and each dyad. The spectral causality components (Equation (9)) are then calculated for all repetitions of each motor task and then averaged over all dyads.

**FIGURE 4:**
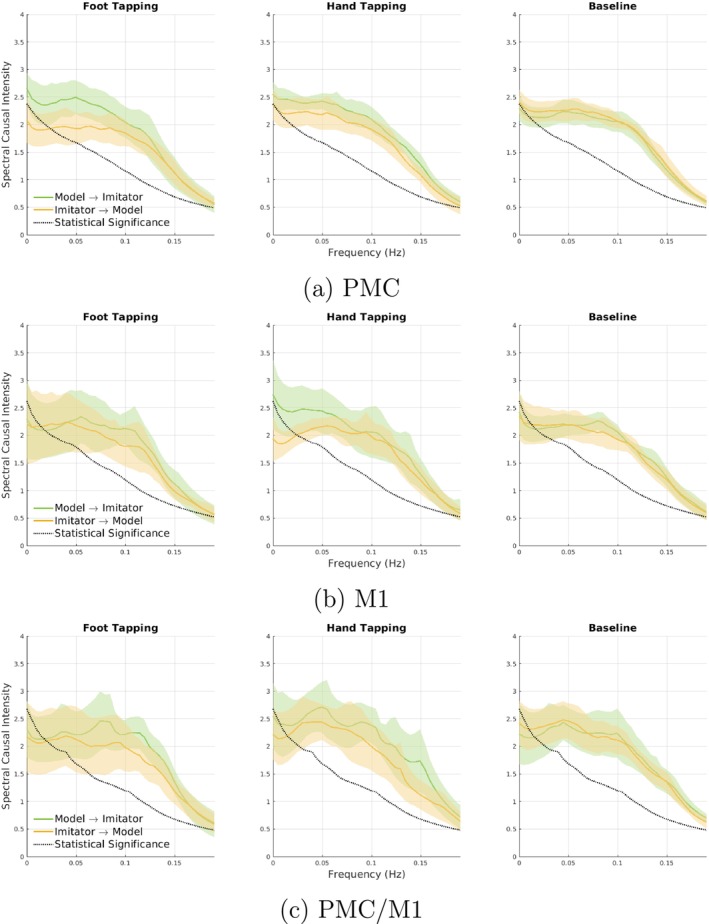
The average and 95*%* confidence interval of the spectral causality of the normalized HbO time series from Model to Imitator (green plots) and from Imitator to Model (orange plots). The average is calculated for each type of event of all dyads for regions: (a) Premotor cortex (PMC), (b) Primary motor cortex (M1), and (c) PMC/M1. The statistical significance is shown in the dotted black line, which is the average spectral causality of the frequency domain surrogate data. Only spectral causality values higher than this line are considered statistically significant.

In all our experiments, time series were first normalized. We then used the MATLAB toolbox for the Spect‐MIR method of Faes et al., (Faes et al. 2022) to calculate the different components of spectral causality as detailed in Section 2.5.1. The model order p was estimated for each trial using the Minimum Description Length (MDL) criterion (Rissanen 2010). We set the maximum model order to max(p)=2 for the state‐space model to be able to capture the short delay between Model and Imitator, which could be as low as one or two samples only (100‐200 ms). Experimental results for the spectral group causality analysis using the Spectr‐MIR method and for different brain regions are shown in Figure 4. These results indicate that the average spectral causality from Model to Imitator is higher than from Imitator to Model in hand tapping and foot tapping in almost all brain regions of interest. In contrast, we see almost equal spectral causality in both directions in baseline intervals of all brain regions. The statistically significant spectral causality in both directions during all intervals can be attributed to the confounding effect of measurement and physiological processes in all intervals. The causal intensity at a specific frequency can be measured as defined by Equation (13) or directly from the difference between the green and orange lines.

Region‐wise, we can notice in Figure 4 that the hand tapping intervals have the highest average causal intensity (the area between the two orange and green curves) in the M1 region, while the causal intensity due to the foot tapping task is the highest in the PMC region. This difference in causal intensity between regions could be because the M1 covers more lateral parts and the hand region is better represented in the lateral areas than the foot region (Metman et al. 1993), while the PMC better represents the foot activities (Cockx et al. 2023).

**FIGURE C1:**
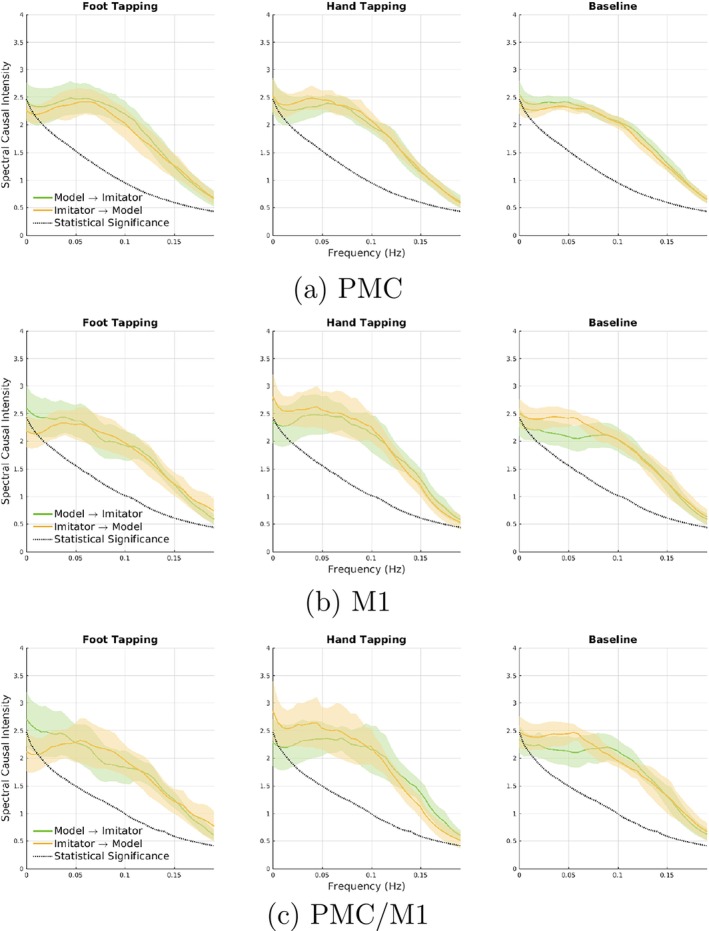
The average and 95*%* confidence interval of the spectral causality of the normalized HbR time series from Model to Imitator (green plots) and from Imitator to Model (orange plots). The average is calculated for each type of event of all dyads for regions: (a) Premotor cortex (PMC), (b) Primary motor cortex (M1), and (c) PMC/M1. The statistical significance is shown in the dotted black line, which is the average spectral causality of the frequency domain surrogate data. Only spectral causality values higher than this line are considered statistically significant.

Spectral causality results for the HbR signals are shown in Figure C1. In contrast to HbO results, the average spectral causality from the Model to the Imitator is only higher than that from the Imitator to the Model in the foot tapping task.

### 3.2 Time Domain Causal Analysis Results

In this section, we compare the Accuracy of estimating the direction of influence using MIR, the time domain Spectr‐MIR (Section 2.6.1), with the time domain group causality baseline methods described in Section 2.6.2: Trace (Zscheischler et al. 2011), Vanilla PC (Janzing et al. 2009), MC‐VAR (Ashrafulla et al. 2013), and 2GVecCI (Wahl et al. 2023).

The time domain causal intensity and direction for the Spectr‐MIR using the integral of Equation (14) is calculated to include only statistically significant values in the frequency range 0.008–0.08 Hz. The statistical significance for each dyad was estimated using the frequency surrogate data method described in Section 2.5.3, averaged over 10 surrogates. To evaluate the performance of all time domain group causal analysis methods, we calculated the Accuracy. For motor tasks, Accuracy is the ratio of intervals where the causal link from the Model to the Imitators is correctly detected. For baseline intervals, Accuracy is the ratio of intervals where the absence of causal link is correctly detected.

**FIGURE 5:**
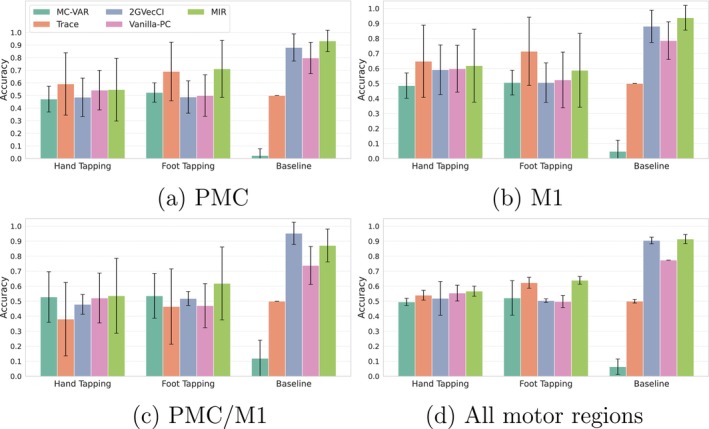
Accuracy (mean ±0.5 std) of the time domain cause–effect analysis of the normalized HbO time series using the time domain method MIR in comparison with four baseline methods: Vanilla‐PC (Janzing et al. 2009), 2GVecCI (Wahl et al. 2023), Trace (Zscheischler et al. 2011), and MC‐VAR (Ashrafulla et al. 2013) for the three different regions: (a) premotor cortex (PMC), (b) primary motor cortex (M1), and (c) PMC/M1; panel (d) shows the mean ±0.5 std of the Accuracies of the three motor regions. The significance level value for the conditional independence test‐based methods is p≤0.05.

Results using HbO time series are shown in Figure 5 for the brain regions PMC, M1, and PMC/M1, along with the average Accuracy across all these motor regions. For the hand tapping task, the best result of the MIR method is for the M1 region (Accuracy= 62*%*), and the worst is for the PMC region (Accuracy= 0.55*%*). Conversely, for the foot‐tapping task, the best results of MIR are from the PMC region (Accuracy= 71*%*), and the worst is from the M1 region (Accuracy= 63*%*). These ROI‐wise results of the MIR method are consistent with similar differences in spectral causal intensity results in PMC/M1 and M1 regions, as discussed in Section 3.1. For baseline intervals, the absence of causality is best detected in the M1 region (Accuracy = 93*%*) and worst in the PMC/M1 region (Accuracy = 88*%*).

On average, Accuracy is higher for the foot‐tapping task than the hand‐tapping task. The higher Accuracy for foot tapping is probably due to a longer delay in HbO activation between the Model and the Imitator. As noted earlier, Figure 2 shows that the reaction time is longer for the foot than for the hand. This longer delay, which can also be seen in Figure 3, made it easier for causality methods to detect who leads (the cause) and who follows (the effect).

The MIR method demonstrates comparable or superior Accuracy to other time domain methods during motor task intervals, as shown in Figure 5. While the Trace method shows higher accuracy in the M1 region for motor tasks, it also exhibits a higher rate of false positives, incorrectly identifying causality during baseline intervals. The high Accuracy or low false positives of the proposed causal intensity measure of MIR, based on the subtraction of the Spectr‐MIR spectral causality $f_{X_{I}\to X_{M}}(\omega )$ from $f_{X_{M}\to X_{I}}(\omega )$, allowed the removal of spurious causal effects that could be attributed to measurement artifact or physiological processes.

Time domain causal analyses of the deoxygenation time series (HbR signals) are presented for all methods in Appendix D. The MIR method showed about 10*%* drop in Accuracy compared to that of HbO signals. This finding is consistent with the MIR method's reliance on a stable temporal relationship between the Model and Imitator, a relationship that, as depicted in Figure B1, is less consistent for HbR than for HbO signals. Despite this, the MIR method's average Accuracy for HbR signals remains comparable or higher than other baseline methods. Finally, similar to HbO results, all methods yield, on average, higher Accuracy for the foot compared to the hand tapping task.

### 3.3 Discussion

Spectral and time domain causal analysis of HbO time series showed that causality can be accurately derived from fNIRS' hyperscanning of a motor task. Our causality measure of MIR achieved 62*%* and 71*%* Accuracies in detecting the causal influence from Model to Imitator in hand and foot motor task intervals, respectively (Figure 5d). Moreover, the proposed causality measure significantly reduced the false positive rate in baseline intervals compared to time domain baseline causality methods. This reduction indicates that the proposed measure enabled the removal of spurious causal effects that might result from task repetition, measurement, or physiological processes. The feasibility of detecting causality in fNIRS based on spectral causal analysis (MIR) was above chance and higher than the Accuracy of four other tested time domain methods. The average spectral cause–effect plots in Figure 4 showed a higher causality from the Model to Imitator than Imitator to Model for hand‐ and foot‐tapping for all regions of interest. Our paradigm was not ambiguous regarding who leads and who follows in the interaction. It was consistent with 10 well‐defined onset blocks of a standardized motor test, typically evoking large responses. The motor imitation task was deemed a good testing paradigm, as it allows for valid control of causality, as only the Model saw the instruction, the Imitator's movement was dependent on the Model. The extracted HbO time series supported the validity of the task: The motor tasks led to an evident signal rise in the expected brain areas. We see evident activation of the averaged HbO time series during the motor tasks compared to baseline (Figure 2).

We observed a clear negative association between HbO and HbR signals Figure 2 during motor task intervals, consistent with findings in fNIRS research under conditions of neural activation (Kinder et al. 2022). This inverse relationship reflects the typical hemodynamic response, where increased neural activity leads to a rise in HbO and a concurrent drop in HbR due to neurovascular coupling and increased cerebral blood flow. Hence, this negative association proves that the observed blood flow changes (HbO, HbR) result from neural activation during motor task intervals.

**FIGURE D1:**
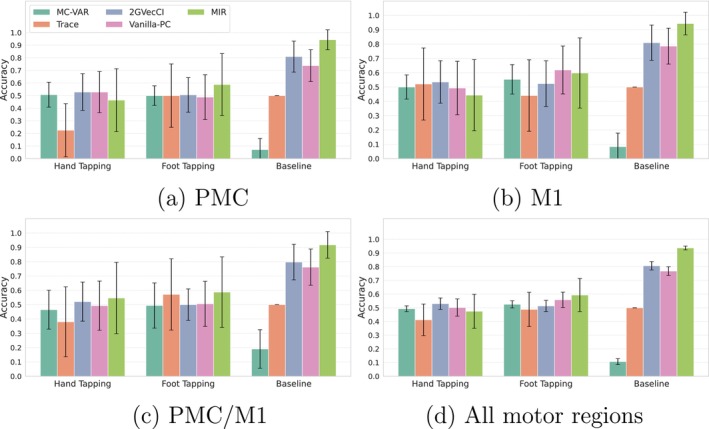
Accuracy (mean ±0.5 std) of the time domain cause–effect analysis of the normalized HbR time series using the time domain method MIR in comparison with four baseline methods: Vanilla‐PC (Janzing et al. 2009), 2GVecCI (Wahl et al. 2023), Trace (Zscheischler et al. 2011), and MC‐VAR (Ashrafulla et al. 2013) for the three different regions: (a) premotor cortex (PMC), (b) primary motor cortex (M1), and (c) PMC/M1; (d) shows the mean ±0.5 std of the Accuracies of the three motor regions. The significance level value for the conditional independence test‐based methods is p≤0.05.

Figure 3 clearly showed the delay in HbO activation of the Imitator relative to the Model, with the Model HbO activation consistently preceding that of the Imitator. In contrast, the HbR signal had less consistent temporal dependencies between Model and Imitator(Figure B1) in Appendix B and consequently, about 10*%* drop in Accuracy compared to the HbO signals (Figure D1).

Overall, the findings of this study contribute to the ongoing debate about the nature and detectability of causality in interbrain dynamics. While Novembre and Iannetti (Novembre and Iannetti 2021) argue that hyperscanning alone cannot provide definitive causal evidence—due to its inherently correlational nature—our results demonstrate that analytical approaches still yield meaningful insights into the directionality of interpersonal neural influence, especially under well‐controlled experimental conditions. In contrast to naturalistic paradigms, our motor imitation task provided a clear causal structure: Only the Model received the instruction, and the Imitator's behavior was contingent on the Model's actions. This unidirectional setup allowed us to validate the performance of our method against a known ground truth.

As Holroyd (Holroyd 2022) critically points out, the field of IBS research is currently challenged by a lack of conceptual clarity, theoretical grounding, and methodological rigor. Our study addresses some of these concerns by explicitly operationalizing causality in a controlled setting and applying a methodologically robust spectral domain approach. While our findings do not challenge the necessity of interventional methods such as dual‐brain stimulation (DBS) (Novembre and Iannetti 2021) for establishing mechanistic causality, they highlight the complementary role of data‐driven causal discovery in advancing the field. When applied to structured paradigms with known causal direction, analytical methods can serve as a bridge between purely correlational hyperscanning and experimental manipulation. In this sense, our work aligns with Holroyd's call for more rigorous operationalization and with Novembre and Iannetti's emphasis on the need for causal testing.

Future work may build on our method to explore its applicability in more naturalistic settings, where causal direction is less constrained and signal‐to‐noise ratios are lower. Moreover, integrating our approach with interventional designs, such as DBS or neurofeedback during naturalistic settings (Gvirts Provolovski and Perlmutter 2021), also referred to as hyperfeedback, could help move the field toward a more mechanistic understanding of IBS and its role in social interaction. In hyperfeedback, feedback is derived from a shared neural parameter (e.g., IBS) across two or more individuals, requiring coregulation of neural activity to achieve a common goal. In DBS, neural oscillations in two individuals are exogenously modulated via transcranial Alternating Current Stimulation (tACS), which allows researchers to assess whether synchrony facilitates social interaction. This method flips the traditional logic of hyperscanning: Instead of observing synchrony as a consequence of social interaction, synchrony becomes the independent variable whose effects on behavior are measured.

A hyperfeedback or DBS study based on the causal inference framework presented here could serve as a controlled intervention paradigm. It would allow researchers to test whether modulating interbrain synchrony causally affects social behavior. This would directly address the call for interventional evidence in the causality debate, help bridge the gap between correlational hyperscanning and mechanistic models of social interaction, and provide a promising path forward in the ongoing causality debate in IBS.

## 4 Conclusion

This study aimed to test whether the direction of influence in dyadic interaction can be derived from fNIRS hyperscanning. To this end, we amended the frequency domain MIR decomposition frameworks of Geweke (Geweke 1982) and Faes et al., (Faes et al. 2022) to fNIRS data of the motor imitation task. We then defined a measure for the direction and intensity of neural influence in frequency and time domains and compared the performance of this measure with four state‐of‐the‐art time domain causal analysis methods. Our study showed that detecting the direction and intensity of neural influence is feasible based on fNIRS data. However, it should be noted that the generalizability of the results of this study is limited by the small sample size of 100 trials for each motor task. The usability of the proposed approach in a natural or uncontrolled setting might also face new challenges that need further investigation. The varying magnitude and temporal delay in HbO activation in response to different tasks make it more challenging for the causal discovery methods to detect the correct causal effect patterns in a natural setting where several motor or nonmotor tasks might co‐occur. We argue that using multimodal imaging or different sources of information is vital for causal discovery in fNIRS hyperscanning. Including emotional influence analysis using facial expressions in dyadic interaction (Shadaydeh et al. 2021), body‐part‐tracking, or verbal signal analysis as additional synchronization measures might support the validation of hyperscanning measurements‐based causal discovery results.

## Author Contributions

**M.S., M.F., I.C., V.N., T.C.:** Conceptualization. **M.S.:** Methodology (Causal and Statistical Analysis). **M.F., I.C., T.C.:** Methodology (Psychology). **M.S.:** Software. **M.S.:** Formal analysis. **T.C., V.N.:** Investigation. **V.N., M.F., MS:** Data curation. **M.S.:** Visualization. **M.S., V.N., M.F.:** Writing ‐ original draft. **I.C.:** Project administration. **I.C., J.D.:** Supervision. **I.C., J.D., M.S.:** Funding acquisition. **M.S., M.F., I.C., V.N., J.D.:** Writing ‐ review and editing. **I.C., J.D.:** Resources. **M.S., M.F., I.C., V.N., J.D.:** Validation.

## Conflicts of Interest

The authors declare no conflicts of interest.

## Peer Review

The peer review history for this article is available at https://www.webofscience.com/api/gateway/wos/peer‐review/10.1111/ejn.70252.

## Data Availability

It should be noted that this study was not preregistered as recommended in (Schroeder et al. 2023). Nevertheless, our data are publicly available on https://osf.io/n4jqc/, and the MATLAB code for data preprocessing is added in E to ensure transparency. For all causal inference methods, we used the publicly available code of the given references with the parameters detailed in Section 3.

## Appendix E Matlab Code for Data Preprocessing

### E.1

data_nirs = SnirfClass([pname, fname]); %read snirf file 'pname/fname'

acquired = data_nirs1;

intensity_nirs = acquired.data.copy;

dod = hmrR_Intensity2OD(intensity_nirs);

% Detect Motion Artifacts

% PARAMETERS:

tMotion = 1;

tMask = 1.0;

STDEVthresh = 10;

AMPthresh = 1.00;

[tIncAuto, tIncAutoCh] = hmrR_MotionArtifactByChannel(dod, acquired.probe,

[], [], [], tMotion, tMask, STDEVthresh, AMPthresh);

%Correct Motion Artifacts

dodMC = hmrR_MotionCorrectSpline(dod.copy, [], tIncAutoCh, 0.99);

%Convert Optical Densities to Hemoglobin Concentration Changes

dhb = hmrR_OD2Conc(dodMC, acquired.probe, [1,1]);

% Bandpass Filtering

dhbFilt = hmrR_BandpassFilt(dhb, 0.008, 0.2);
